# Feature tracking in cardiac magnetic resonance imaging to evaluate normal myocardial function

**DOI:** 10.1186/1532-429X-15-S1-E51

**Published:** 2013-01-30

**Authors:** Sebastian Buss, Philipp Matheis, Kristin Breuninger, Rebekka Kammerer, Yannick Sander, Birgit Krautz, Lukas Rust, Christian Galuschky, Grigorios Korosoglou, Evangelos Giannitsis

**Affiliations:** 1 University of Heidelberg, Heidelberg, Germany; 2 TomTec Imaging Systems, Munich, Germany

## Background

Assessment of left ventricular function (LV) is one of the most important tasks of clinical cardiac magnetic resonance imaging (CMR). Regional and global LV function has been recognized to differentiate various myocardial disorders. The aim of the study was to provide normal values for myocardial deformation parameters derived from the feature tracking imaging (FTI) algorithm applied to standard CMR cine SSFP sequences in a large group of healthy subjects.

The feature tracking algorithm (2D CPA MR©, TomTec Imaging Systems GmbH), is a two dimensional deformation analysis of the myocardium that was originally designed for echocardiographic image analysis, which has now been transferred to CMR SSFP sequences without the need for additional scans. This novel approach may have potential advantages over existing methods, such as broad availability, vendor independency and lack of time-consuming post processing.

## Methods

Circumferential, radial and longitudinal peak systolic endocardial regional and global strain values of 110 healthy subjects (53 women and 57 men, mean age 48 ± 12 years) were measured using FTI within a standard protocol in a 1.5T CMR-scanner. FTI was applied to standard SSFP sequences. The strain values were derived from three cine long and three short axis views.

## Results

Global peak systolic radial strain was 35 ± 8%, global peak systolic circumferential strain was -27 ± 4% and global peak systolic longitudinal strain was -24 ± 4%. In younger subjects (<43 years) versus older subjects (>58 years) radial (34 ± 7% vs. 34 ± 7%), circumferential (-27 ± 3% vs. -28 ± 4%) and longitudinal (-25 ± 4% vs. -23 ± 4%) strains were not significantly different. Women had higher levels for circumferential and longitudinal strain in comparison to men whereas radial strain was lower (p<0.05).

## Conclusions

Our study provides for the first time normal values derived from a large cohort of healthy subjects for cardiac deformation analysis with CMR using a feature tracking algorithm. Without performing any additional scans, FTI is feasible and provides the possibility for a reliable, objective and fast quantification of LV regional and global function. In summary, this novel method may have great importance in clinical routine and trials using CMR and provide further insight into LV function.

## Funding

None.

